# Insights into midgut cell types and their crucial role in antiviral immunity in the lepidopteran model *Bombyx mori*

**DOI:** 10.3389/fimmu.2024.1349428

**Published:** 2024-02-14

**Authors:** Mian Muhammad Awais, Shigang Fei, Junming Xia, Min Feng, Jingchen Sun

**Affiliations:** Guangdong Provincial Key Laboratory of Agro-Animal Genomics and Molecular Breeding, College of Animal Science, South China Agricultural University, Guangzhou, China

**Keywords:** columnar cells, goblet cells, regenerative cells, endocrine cells, immunity, peritrophic membrane, digestive juice

## Abstract

The midgut, a vital component of the digestive system in arthropods, serves as an interface between ingested food and the insect’s physiology, playing a pivotal role in nutrient absorption and immune defense mechanisms. Distinct cell types, including columnar, enteroendocrine, goblet and regenerative cells, comprise the midgut in insects and contribute to its robust immune response. Enterocytes/columnar cells, the primary absorptive cells, facilitate the immune response through enzyme secretions, while regenerative cells play a crucial role in maintaining midgut integrity by continuously replenishing damaged cells and maintaining the continuity of the immune defense. The peritrophic membrane is vital to the insect’s innate immunity, shielding the midgut from pathogens and abrasive food particles. Midgut juice, a mixture of digestive enzymes and antimicrobial factors, further contributes to the insect’s immune defense, helping the insect to combat invading pathogens and regulate the midgut microbial community. The cutting-edge single-cell transcriptomics also unveiled previously unrecognized subpopulations within the insect midgut cells and elucidated the striking similarities between the gastrointestinal tracts of insects and higher mammals. Understanding the intricate interplay between midgut cell types provides valuable insights into insect immunity. This review provides a solid foundation for unraveling the complex roles of the midgut, not only in digestion but also in immunity. Moreover, this review will discuss the novel immune strategies led by the midgut employed by insects to combat invading pathogens, ultimately contributing to the broader understanding of insect physiology and defense mechanisms.

## 1 Introduction

**Figure 1:**
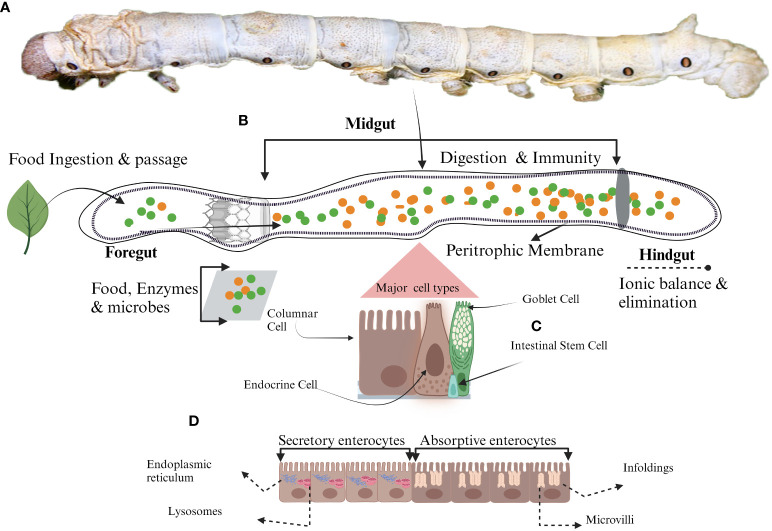
Schematic representation of midgut cell types. **(A)** Mature larvae, **(B)** The midgut, **(C)** Different types of midgut cells in silkworm **(D)** Different roles of enterocyte due to the presence of various organelles. Secretory enterocytes in anterior midgut contain secretory organelles such as endoplasmic reticulum and lysosomes whereas, absorptive enterocytes located in the posterior midgut have absorptive organelles like microvilli and infoldings to support the absorption of the nutrients.

In insects, the gastrointestinal tract, despite variations in its morphology (due to differences in feeding habits), comprises a monolayer of epithelial cells surrounded by visceral muscles. This intricate anatomical structure is divided into three discrete regions - the fore, mid, and hindgut (**Figure 1**), each characterized by distinct attributes, roles, and embryonic origin (1). In *Drosophila* and silkworms, the midgut is a complex tissue bearing a striking resemblance to its mammalian counterpart (2). Its multifaceted functions include digestion, immune responses, the regeneration of the aged cells, and recovery of the infected luminal tract.

The second-largest organ within the insect body is involved in digestion-related activities and holds critical importance in the insect’s defense against pathogens (3). It serves as the initial line of defense against invading pathogens. One of the most conspicuous aspects of the midgut’s immune function lies in its ability to produce antimicrobial peptides (AMPs) and reactive oxygen species (ROS) (4, 5). These molecular weapons are integral components of the insect’s defense arsenal to combat and neutralize potential threats from invading pathogens. The production of AMPs is a particularly significant aspect of this defense mechanism, as these small yet potent molecules possess the ability to target a wide array of invading microorganisms (4). Researchers have dedicated significant efforts to unravel the intricate mechanisms underlying AMP production and to isolate novel AMPs (6).

Beyond its role in AMP production, the midgut also plays a crucial part in maintaining the intricate balance of gut microbes. To maintain this delicate equilibrium, the midgut expels disease-related microbes and pathogens (7), preserving a beneficial microbial community. The unique immunological features of the midgut render it a subject of considerable scientific interest and investigation. This ongoing pursuit of knowledge concerning the midgut’s immune functions is invaluable, as it contributes to a deeper understanding of silkworm’s immunity. Ultimately, these insights not only enhance our comprehension of silkworm biology but also hold the potential to inform broader discussions on immunity across various biological systems.

**Figure 2:**
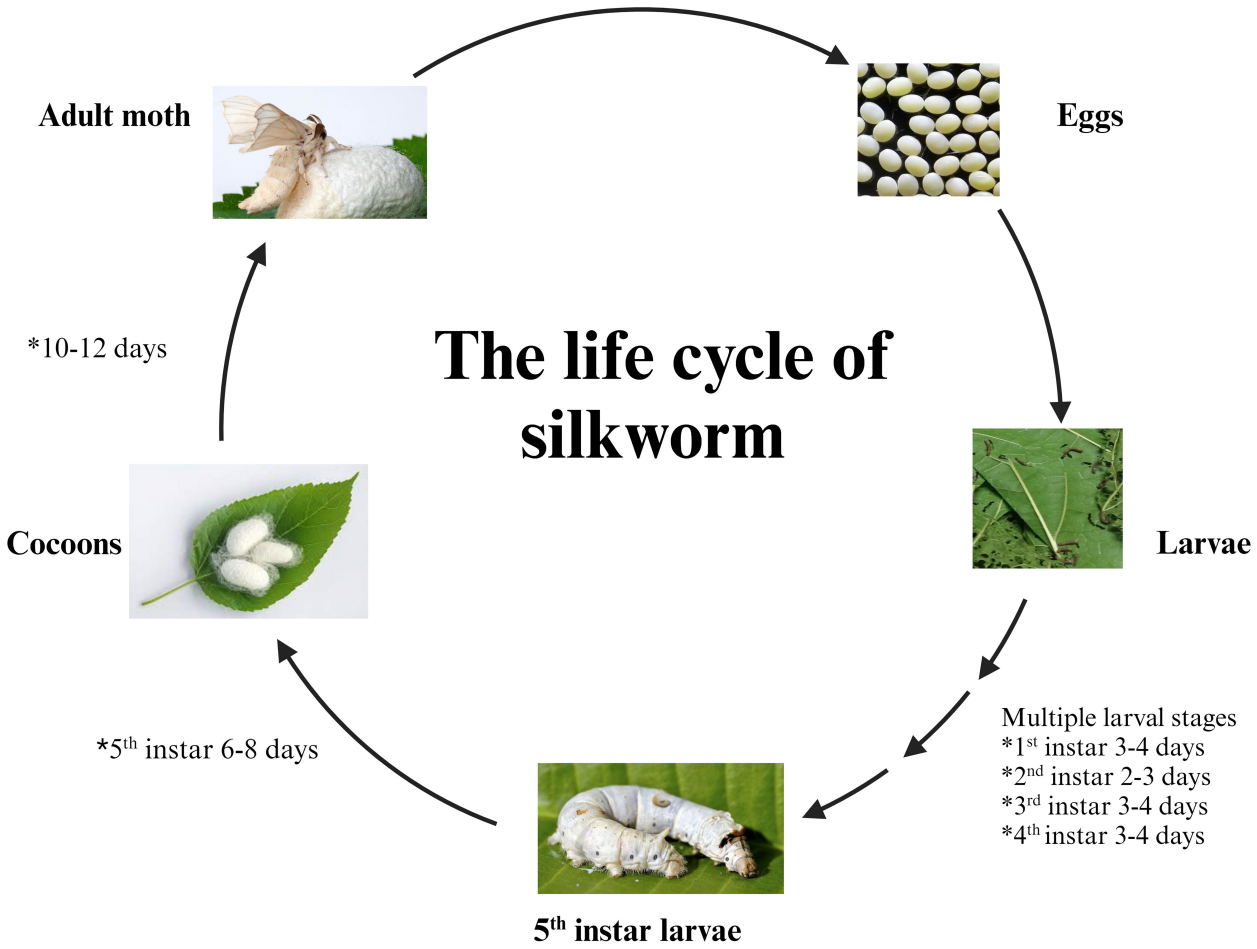
The Silkworm *(B.mori)*: from egg to adult.

Further, the utilization of *Bomby mori* as a model organism offers numerous advantages for scientific research (8). Notably, a straightforward and well-characterized genome sequence has facilitated genetic manipulations and studies on gene functions and regulations (9). The short life cycle, progressing through stages in a matter of weeks and big progeny size, enables rapid experimentation and observation (**Figure 2**). Silkworms are easy to handle and can be maintained in laboratory conditions with a simple diet of mulberry leaves. Additionally, the large size of silkworm eggs and the transparency of their pupal stage enhance experimental accessibility (10). Their well-defined developmental stages, shared biological processes with other insects, and certain similarities to mammals further elucidate the value of silkworms as versatile model organisms across various research disciplines (11).

### 1.1 Modern-omics technologies and cell types

The advent of single-cell RNA sequencing technology (scRNA-seq) has revolutionized our understanding of gene regulation at the single-cell level by scrutinizing the gene expression profiles of thousands of individual cells, thus facilitating the identification of previously unknown cell types and their unique physiological states (12). Moreover, it has provided a means to unravel the intricate developmental trajectories of cells, shedding light on their origins and evolutionary pathways (12). Recent transcriptomic investigations have unveiled different cell types in the midgut in *Drosophila* (2, 13) mosquitoes (14) and silkworms (15), and a compelling narrative, revealing that the insect midgut, exemplified by *Drosophila* and silkworms, closely related to the mammalian small intestine in terms of the cell types present and their associated marker gene expression patterns (2). **Table 1** summarizes well-established cell type markers from scRNA-seq analysis of midgut tissues in *Drosophila*, mosquitoes, silkworms and mice. In the model insect *Drosophila*, the midgut undergoes renewal at regular intervals of one to two weeks, facilitated by specialized regenerative cells known as intestinal stem cells (ISCs) (17). These ISCs exhibit similarities with mammalian transit-amplifying cells (TACs) in the G2 phase of the cell cycle. Additionally, within the midgut milieu in insects, there are endocrine cells, enterocytes, and enteroblasts, all of which share akin marker gene expression patterns when compared with their counterparts in mammals (2). In *Drosophila*, enteroendocrine cells (EEs) mirror their mammalian counterparts, while goblet cells found in lepidopteran midguts resemble mammalian goblet cells. Furthermore, cardia cells within the *Drosophila* midgut exhibit marked similarities in marker expression profiles with the goblet cells of mammals (2). Similar observations were reported by Xia et al. in a recent study, wherein diverse cell types identified in the silkworm midgut exhibited nearly identical patterns of marker gene expression (15). This convergence extends to the functional domain, where homologous marker genes underscore numerous facets of midgut function. These findings shed light on the remarkable conservation and convergent evolution of midgut biology across diverse taxa, underscoring its significance in understanding insect physiology and potential implications for our comprehension of mammalian gastrointestinal biology.

**Table 1:** Marker genes for major cells types in mice, *Drosophila* and *Aedes aegypti*.

Organism	Mice		*Drosophila*		*Aedes aegypti*		*Bombyx mori*	
Cell types	Potential marker	Reference	Potential marker	Reference	Potential marker	Reference	Potential marker	Reference
Columnar cells (Enterocytes)	Reg3g, Gsdmc4, Prss32, Krt8, Krt19, Reg3b, Gsdmc2, Apoa1, Mep1b	(16)	Trypsin genes, Alpha-theta trypsin, Lab, Vha100-4	(2)	Nubbin, Ubiquitin, Trypsin, Aquaporin, Lipase, V-type proton ATPase	(14)	Ara, Amy35, Bs,	(15)
Endocrine Cells	Chgb, Chga, Gfra3, Trp53i11, Neurod, Vwa5b2, Cpe,T ac1,Fam183b	(16)	Pros, AstA, NFP, AstC	(2)	Prospero,	(14)	Npfr-a4, Npf, Npy2r-a10, Npfr-a11	(15)
Intestinal Stem cells (Tansit amplifying cells in Mice model)	Hspd1, Ptbp1, Snora26, Snhg20, Rps271, C1qbpwdr4, Nxt1, Ppil1, Rfc2	(16)	Delta, Esg	(2)	*Delta*, *Klumpfuss*	(14)	NID1,	(15)
Goblet cells	Clca3,Zg16, Fcgbp,Tff3, Agr2,Scin, pdia5, Spink4, Muc2, Ccla6Klk1	(16)					Ets98B	(15)

Regarding the origin of different types of cells, recent studies have revealed that the self-renewable ISCs similar to TACs in mammals raise enteroblasts (EBs) or enteroendocrine progenitor cells (EEPs), and the notch signaling pathway controls this determination. Heightened notch signaling pathway directs ISCs toward a developmental trajectory culminating in the formation of absorptive enterocytes (ISC → EB → EC) (18, 19). In contrast, when notch signaling is maintained at a lower level, ISCs follow an alternative route, leading to the generation of hormone-secreting enteroendocrine cells (ISC → EEP → EEs) (20). This finely regulated process of cellular fate determination plays a critical role in regulating the composition and functional dynamics of the insect midgut.

This review will discuss a concise yet comprehensive overview of the cell-level functionalities of the insect midgut, with a particular focus on the silkworm, from the insights derived from advanced transcriptomic studies. Additionally, we discuss the role of various midgut cell types in the immune defense mechanisms against pathogens. This integrated approach aims to provide readers with a coherent and deep understanding of the immunologically active organ, the insect midgut.

## 2 Mid-gut cell types

### 2.1 Columnar cells/enterocytes

Columnar cells (CCs) are pivotal among the four primary cellular phenotypes within the insect midgut (**Figure 1**) (7). They are referred to as “enterocytes” by the feature of their akin absorptive functionalities to their mammalian small intestinal counterparts (21–23). Beyond their absorptive nature, these CCs prominently orchestrate the synthesis and secretion of digestive enzymes, thereby underscoring their indispensable contribution to digestive processes within the insect midgut environment (24). The distinct anatomical feature of CCs includes the centrally located nucleus accompanied by a prominently convoluted apical membrane, giving rise to microvilli with an inherent actin cytoskeletal structure (**Figure 1**) (25). In addition to the common characteristics inherent to CCs across insect species, there exist specialized ultrastructural features that are discernible within distinct regions of the midgut and feeding behaviors of insects (26–29). The ultrastructural composition of CCs within the midgut exhibits a transition along the longitudinal axis, displaying distinct roles in the shifting anatomical context (21). Structural evidence highlights that CCs in the anterior midgut are responsible for secreting an array of digestive enzymes encompassing exopeptidases, maltases, endopeptidases, amylases, lipases, and lysozymes (**Figure 1**) (24, 30). These enzymes are secreted into the midgut lumen via conventional exocytosis and through mechanisms involving apocrine or micro-apocrine modes of secretion (30). Conversely, CCs within the posterior midgut region are characterized by their involvement in synthesizing serine proteases (31–33). Given that digestive enzymes play a direct and crucial role in facilitating nutrient accessibility and development (34, 35), the production and activities of these enzymes are regulated by a rigorous regulatory framework. Multiple factors, encompassing the composition of nutrients, signals from endocrine and neuronal sources, and interactions with gut microorganisms, collectively contribute to the intricate modulation of enzymatic processes underlying nutrient digestion and absorption (36–39). Nutrient absorption within CCs is executed through the activity of transport proteins, which exhibit distinct distribution patterns upon the regionalization of the midgut (**Figure 1**) (32, 40). The Na^+^/K^+^ ATPase channel for absorption is absent in CCs, and the cotransporters of CCs capitalize on the electrochemical gradient of K^+^ rather than that of Na^+^ as a driving ion. The amino acids from protein digestion are translocated from the lumen into the intracellular cytoplasm, marking the specialized functionality of CCs in nutrient absorption (41, 42).

#### 2.1.1 Role of CCs in immunity

CCs play a multifaceted role in immunity and nutritional uptake. These cells secrete enzymes that have been demonstrated to have a significant role in immunity within various insect species. For instance, the lysozymes secreted by CCs regulate the microbial load across distinct midgut regions in synergy with the alkaline environment (43). A myriad of proteases is secreted into the lumen of the insect gut by CCs. Alongside their primary function of enzymatic breaking down of ingested food, they also play a critical role in degrading proteins linked to invading pathogens. Importantly, these proteases possess the ability to target the proteins of such pathogens. This includes the structural components of virions and their surface proteins crucial for initiating cellular infection (44, 45). Bm-SP142 is a 35 kDa protease mainly expressed in the middle part of the silkworm midgut, effectively impairs *Bombyx mori* nuclear polyhedrosis virus (BmNPV)’s ability to infect BmN cells. Furthermore, Bm-SP142 has demonstrated its efficacy in diminishing the propagation efficiency of both BmNPV and *Bombyx mori* bidensovirus (BmBDV) within silkworms, indicating the antiviral potential of proteases secreted by CCs (46). It was reported that a midgut serine protease, the alkaline digestive enzyme trypsin termed Alkaline A (BmTA), has antiviral potential against BmNPV. The proteomic analysis elucidated a substantially high level of BmTA in the resistant silkworm larvae compared to the susceptible. Viral analysis showed a decreased level of the Vp39 gene in the recombinant-BmTA-treated group (47).

The secretion of the signaling protein Hedgehog has been well-documented in both *Drosophila* and *B. mori* (48, 49). Following infection by *Escherichia coli* or *Bacillus thuringiensis*, a notable proliferation of midgut cells and upregulation of Hedgehog genes have been observed in the silkworm. When genes associated with the Hedgehog pathway were experimentally silenced, cell growth was suppressed, indicating the pathway’s regulatory role (49). These findings provide insights into the critical role of signaling proteins in immunity against pathogens. Notably, in *Drosophila*, these proteins exhibit a connection with nutritional availability and developmental process as these play a role in the delayed pupation observed in starved flies, attributed to their capacity to inhibit ecdy-streroid production due to a reduction in the transcription of genes linked to enzyme production related to molting (48).

Given the direct correlation between nutrient availability, growth, and development, which collectively underpin animal survival (50–52), it becomes evident that CCs from the midgut engage in communication to synchronize responses and evade the deleterious effects of invading pathogens. This interplay serves as a strategic mechanism to counteract the pathogenic influences. CCs’ the crucial components of midgut epithelium, have a dynamic role in insect health and survival, serving as a critical nexus between digestion and immunity.

### 2.2 Endocrine cells/Enteroendocrine cells

Nutrient uptake and digestion are the critical functions performed by the insect midgut. Along with these vital physiological phenomena’s essential for growth and development, another critical function performed by the gut is the maintenance of gut homeostasis. The homeostasis is maintained through the complex gut-brain axis, forming a neurohumoral communication system. Thanks to midgut EEs, which secrete biologically active peptides responsible for the sophisticated interplay of communication within the organism (38, 53, 54). **Table 2** summarize some important peptides secreted by the EEs. EEs discovered around three decades ago (64–66), have two distinct morphological forms, open and closed types (67), characterized by their peptidergic nature (64); the former have direct contact with the midgut lumen (67, 68), while the latter lacks such interaction as they do not extend through the epithelium (**Figure 1**) (33, 67). The functional application of recent genomic and proteomic approaches provides compelling evidence of the critical role of EEs within the insect midgut play in the physiology and developmental processes of insects (56, 69, 70). The EEs of silkworms were classified into four distinct subtypes according to their spatial distribution. Type I cells were identified across all three midgut regions, while type II, III and IV cells were localized in the anterior, middle, and posterior midgut, respectively (56). The spatial distribution of EEs across different midgut regions also depends on the specific peptide they produce (17, 71). The peptides are transformed into active states by cleavage and post-translation modification from protein precursors produced by EEs. In *Drosophila*, 9 major precursors were identified (58), whereas in silkworms, 18 distinct protein precursors were identified (56). In different insects, the peptides produced after cleavage are mainly involved in the regulation of food in the alimentary canal, from reduced ion transport to suppressed gut contractions (72, 73). In *B. mori* tachykinin peptides (Tk) secretions are regulated by *B. mori* gustatory receptor (*BmGr4*) expressed in some Tk-producing EEs in the anterior midgut when food and digestive products arrived after feeding began (60). The regulatory role of peptides secreted by EEs is evident in locusts and *Drosophila*. Notably, in the midgut of starved locusts, the levels of Tk diminish while their circulation in the hemolymph increases, suggesting a potential responsiveness to nutritional status for enhanced food uptake (74). Further insights have emerged from studies in *Drosophila*, demonstrating that the EE-produced Tks contributes to lipid metabolism through interaction with their receptor TKR99D situated in the brain (61). Targeted ablation of EEs expressing TKs resulted in the suppression of intestinal stem cell proliferation, indicating the developmental role of these peptides (37).

**Table 2:** Peptides produced by endocrine ells and their functions.

Peptide	Produced By	Functions	Reference
Allatostatins	Endocrine cells	JH secretion, AMP production	(55–57),
Diuretic hormone 31	Endocrine Cells	Visceral muscle contraction	(58, 59),
Tachykinin (Tk)	Endocrine cells	Food sensing & Digestion	(60, 61)
Orcokinin	Endocrine cells	Regulation of ecdysis	(62)
Ryamide	Endocrine cells	Feeding & Digestion	(56)
Neuropeptide F	Endocrine cells	Feeding & digestion modulation	(63)
Myosupression	Endocrine cells	Food sensing & gut contractions	(56, 60),
CCHamide	Endocrine Cells	Gut muscle contraction	(17, 56, 58),

#### 2.2.1 Role of EEs in Immunity

Few of the immunologically important peptides produced by the EEs are enlisted in the **Table 2**. Allatostatins, initially identified in cockroaches, are produced by insect brain and midgut EEs (75). The midgut of silkworms displays a pronounced enrichment of allatostatin receptors compared to the brain (76). These peptides play a crucial role in the development and secretion of juvenile hormone (55). Their involvement in immune functions has been documented in the well-studied model organism *Drosophila* (77). In red imported fire ants, an upregulation of allatostatin A2 expression was observed in response to *Solenopsis invicta* virus-3 (SINV-3) infection. The elevated expression of the peptide consequently leads to sharp production of the AMP cecropinA1, serving as an immune strategy (57). The midgut EEs produce another bioactive peptide known as diuretic hormone 31, which exhibits an immune response when faced with bacterial infection. ROS accumulates within the midgut lumen in a manner contingent on the transient receptor potential ankyrin 1 (TRPA1) receptor. This receptor activation enables EEs to release the diuretic hormone and its receptor in the neighboring visceral muscles, initiating the contraction of longitudinal visceral muscles. As a consequence, the expulsion of the pathogen from the midgut is facilitated.

EEs secrete peptides, which are also released from neurons (70, 78), posing a challenge in precisely attributing roles to EEs. Notably, midgut EEs are postulated to release peptides that exert paracrine effects on adjacent cells. While extensive research investigations are done to elaborate the EEs functions in development and immunity using model insects, a significant knowledge gap exists in comprehending the regulatory mechanisms governing EE-derived peptides in other insects, such as silkworms. Molecular and functional analyses utilizing cutting-edge techniques are imperative to elucidate the regulatory role of peptides comprehensively. These investigations can potentially unravel the intricate landscape of midgut physiology and insect homeostasis. Moreover, they offer insights into how the second-largest organ in insects contributes to immune defense against invading pathogens. This pursuit advances our fundamental understanding of endocrine activity from a molecular perspective and sheds light on broader physiological implications.

### 2.3 Goblet cells

Lepidopteran insect’s midgut possesses a distinctive category of cells known as goblet cells (GCs) (21, 23, 79, 80). These GCs, in conjunction with the midgut’s CCs, play a pivotal role in the elevated pH of the midgut environment. These insects primarily feed on plant matter; their food encompasses secondary metabolites, such as tannins and phenols, which can be detrimental. The presence of these secondary metabolites diminishes nutrient accessibility, as they form insoluble complexes via cross-linking with enzymes and proteins. To counteract this constrained nutrient availability, the coordinated efforts of GCs and CCs come into play, preserving an elevated pH within the midgut lumen. GCs are characterized by a conspicuous chalice-shaped central cavity, which originates from the invagination of the apical membrane (**Figure 1**) (21, 22, 81, 82). Their distribution within the midgut varies according to their respective locations, resulting in heterogeneous concentrations (21). These GCs establish intricate connections with other midgut cells, employing septate and gap junction formations to facilitate intercellular communication and coordination (83). The chalice-shaped central cavity (Goblet cell cavity, GCC) is lined by small structures known as microvilli, and these microvilli are longer at the basal portion of GCC than the apical portion, providing extensive surface area. These microvilli are surrounded by actin. The GCC content is effectively separated from the intestinal lumen through a sophisticated apical structure called a valve. This valve consists of densely packed microvilli lacking mitochondria, which do not allow the free movement of the content (82). GCs play a pivotal role in preserving the distinctive characteristic of elevated alkaline pH within the midgut of these insects. This distinct milieu is upheld through the presence of the vacuolar-type proton pump (V-H^+^ ATPase) (82, 84, 85). This pump is actively engaged in the transportation of protons (H^+^) from the cytoplasm into the interior of GCCs (86, 87) at the expense of energy against the concentration gradient (82). Mitochondria located within the microvilli supply the requisite energy for this process. The active translocation of H^+^ is instrumental in the preservation of a transmembrane electrical potential of 150 mV (88). The V-H^+^ ATPase pump orchestrates an intricate exchange between H^+^ and K^+^, resulting in the net flow of K^+^ from the cytoplasm of GCs into the interior of the GCCs (81). This establishes an electrochemical gradient conducive to the efflux of K^+^ from the GCCs into the lumen of the midgut via a specialized valve. This transport phenomenon is augmented by the activity of GCs carbonic anhydrase, which facilitates the generation of a flux of HCO3^-^ (81) This flux-causing K^+^ transport is essential in maintaining the elevated pH within the midgut.

#### 2.3.1 Role of GCs in immunity

GCs within the insect alimentary tract are similar to their mammalian small intestinal counterparts, notably evidenced by large cavities embellished with microvilli (2, 89). The distinctive hallmark of the mammalian intestinal goblet cells is the production of mucus, forming a protective layer. This layer is critical in shielding the intestinal milieu from the abrasive action of ingested food and invading pathogens (90). Similar observations related to mucin secretion and the associated epithelial immune competence have been documented within fishes. Goblet cells encompass a substantial proportion of the epithelial constitution in fish (91). Notably, these cells evince an increase in size following exposure to microplastics (MPs), indicative of escalated mucus production. This phenomenon, in turn, confers enhanced epithelial protection against the abrasive effects exerted by MPs (92). The macromolecular composition of mucous secretions encompasses essential constituents like mucin proteins (93) alongside immunomodulatory proteins (94). This collective evidential paradigm underscores the multifarious contributions of goblet cells and their mucous effluents toward maintaining epithelial integrity and immune homeostasis (95). The histological resemblance observed among the GCs in mammals, fishes, and insects suggests a potential similarity in the functional roles attributed to these specialized insect cells. Several scientific investigations have revealed the significant contribution of GCs to immunity in mammals and fishes. Consequently, a conjecture arises that these cells might potentially serve an immunological role in insects, specifically lepidopteran insects. Despite relatively limited research, studies have begun to shed light on the involvement of GCs in immunity, exemplified by their response in instances such as that observed in *Helicoverpa armigera.* When the larvae of *H. armigera* were subjected to an infection by the cytoplasmic polyhedrosis virus, a typical pathogen targeting the midgut, a visible deformation in the midgut’s structural architecture emerged. This perturbation was accompanied by the emergence of anomalous microvilli projections subsequent to the viral infection (96).The GCs, historically linked with mucin secretion and maintenance of epithelial barrier function, might possess a multifaceted role beyond these established functions. The emergence of analogous histological features across diverse taxa sparks the inquiry into the potential conservation of immune functionalities associated with GCs. Given the intricate interplay between host organisms and pathogens, investigating the role of GCs in insect immunity represents a frontier of scientific inquiry that warrants further investigation. In the context of insects, such as *H. armigera*, the observed response of GCs to viral infection introduces a novel dimension to the understanding of insect immune defense mechanisms. The morphological alterations in the midgut’s microarchitecture following viral challenge allude to the potential involvement of GCs in orchestrating immune responses. Elucidating the precise molecular mechanisms that underpin this phenomenon remains an intriguing avenue for future research endeavors.

### 2.4 Stem cells

The gastrointestinal tract of insects faces numerous challenges, including the abrasive effects of ingested food’s movement along the alimentary canal interactions with gut microbiota, a primary barrier against ingested toxic compounds and invasive pathogens. ISCs of midgut play a pivotal role in these critical functions. ISCs are also critical in upholding gut integrity and homeostasis, even within the challenging environment of the digestive tract of insects (97). ISCs positioned along the basal side of the pseudostratified epithelial monolayer (**Figure 1**) exhibit resemblances to their mammalian counterparts as reported by the Huang et al. in *Drosophila* (2). Functioning as self-renewing and multipotent entities, these cells have the ability to give rise to diverse differentiated cell lineages constituting the intestinal tract (98, 99). ISCs have blast-like morphology characterized by fewer organelles and a cytoplasm of lighter density (22). Notably, recent research has unveiled the presence of storage entities, lipid droplets and glycogen granules within ISCs (100, 101). The division of ISCs depends upon the cellular function performed after division. Two types of cell divisions are observed in ISCs, i.e. asymmetric and symmetric. In the asymmetric division, to maintain the constant population of ISCs within the gastrointestinal milieu, ISCs generate a stem cell and a terminally differentiated counterpart, poised to undertake specific functional roles. In contrast, the symmetric division modality results in the formation of two daughter cells, both of which assume mature and functional phenotypes (40, 102, 103). Once the ISCs are divided and differentiated, they can’t revert to stem cells (104). This interplay between divisional strategies underscores the regenerative prowess of ISCs but also attests to their pivotal role in orchestrating tissue homeostasis and repair.

#### 2.4.1 Indirect involvement in immunity

Various factors are responsible for the depletion of CCs, including gut microbes, pathogenic microorganisms and the production of AMPs as an immune strategy. The study of the model insect *Drosophila* has been instrumental in shaping our current knowledge regarding the involvement of ISCs in both immunity and tissue renewal. The efficient and rapid restoration of the lost cells is accomplished by a coordinated immune response with an epithelial renewal mechanism that facilitates the repair of the damage (37, 105). ISCs proliferation following infection contributes substantively to replenishing the depleted cell populations, with this process being intricately governed within the midgut milieu. The regulation of ISC activities, requisite to rebuild the cellular deficits, is elicited through the involvement of established canonical signaling pathways activated upon pathogenic invasion. Moreover, the phenomenon of epithelial renewal has been discerned as effective in countering the immune effects against oral viral infection (106). Hemocytes could also induce the proliferation of ISCs during systemic infections, resulting in increased epithelial renewal within the gut (107, 108). This process is significant due to its essential role in improved immunity and recovery during such infections. Central to this phenomenon is the increase of gut renewal orchestrated by the release of secreted ligands of the Upd family, which, in turn, engage the JAK/STAT pathway. During oral infections, hemocytes are employed in the midgut, which triggers the release of Dpp, orchestrating the stimulation of ISCs at an early infection phase and restraining this activation through the recovery stage (109). The intricate modulation of these ligands poses a complex regulatory puzzle, necessitating further exploration into the respective roles played by hemocytes, ISCs, and visceral muscles, which also partake in the regenerative cascade (110, 111). Recent studies have begun unrevealing the involvement of signaling pathways and transcription factors underlying the expression of Upd ligands within the midgut (112). The proliferation of ISCs following infection is regulated by diverse signaling cascades mediated by cell-autonomous and non-cell-autonomous mechanisms. Canonical pathways such as JAK-STAT, EGFR (103, 113, 114), and others, including Wnt/Wg (115), BMP (116), Hippo (117), JNK (118), and p38, play crucial roles in orchestrating the proliferative capacities of ISCs. Nonetheless, a comprehensive elucidation of their integrated regulation, ensuring a balanced proliferation of ISCs, remains elusive. Further investigations are necessary to understand these interactions and regulatory networks that underlie the intricate balance governing ISC proliferation, thereby advancing our understanding of intestinal homeostasis in the context of infection.

While ISCs aren’t directly involved in the immune response, their significance is evident in their pivotal role in expelling pathogens from the midgut and initiating the regeneration of the gut’s epithelium, which aids in the recovery of the insect after infection. Despite these insights, many uncertainties persist regarding the precise functions of ISCs in immunity. Considerable research efforts are required to investigate the full potential of these ever-young cells within the midgut in the context of immunity, particularly in lepidopteran models like silkworms. Such investigations promise not only to enhance our comprehension of the involvement of ISCs in immunity and epithelial renewal but also to unveil novel immune strategies employed by this multifunctional organ in insects.

## 3 Peritrophic membrane and immunity

In insects, an acellular structure known as the peritrophic membrane (PM) exists alongside various cell types within the midgut (**Figure 1**) (119–121). The PM, a semi-permeable membrane-like structure (**Figure 1**), is produced by epithelial cells (122). It possesses chitin as an essential component, cross-linked with glycoproteins to form a crucial component of the midgut’s defense and immune system (121). With a thickness ranging from 0.5 to 1.0 µm, the PM plays a pivotal role in selectively facilitating the transport of nutrients and ions while also shielding the midgut cells from abrasion caused by ingested food and potential harm from pathogens (121, 123). The composition of the PM matrix encompasses PM proteins (124), and numerous investigations have reported varying numbers of proteins within the PM matrix of different lepidopteran insects. For instance, there are reports of 305 proteins in silkworms (125) and 41 proteins in *H.armigera* (126) The extent of involvement of these proteins in PM thickness is contingent upon the composition of ingested food. It is hypothesized that these proteins are stored within the midgut cells and are subsequently released for PM synthesis in response to stimuli from ingested food (127). Although the thickness of PM during growth is evident in diverse insect species such as *Ostrinia nubilalis* (128), *Manduca sexta* (129), *Anomala cuprea* (130)*, Tribolium castaneum* (131), *Melipona quadrifasciata*, and *Apis mellifera* (132). This thickening of the PM in response to the stimulus of food is not confined to Lepidoptera; it extends to other insects, such as *Anopheles gambiae*, where the post-blood feeding localization of *Ag-Aper14* within the ectoperitrophic space has been documented (133). However, dietary conditions or starvation have no impact on the mRNA expression levels of peritrophins in *Spodoptera litura* (134) and insect intestinal mucins (IIMs) in *M. configurata* (135). The complex structural characteristics of the PM elucidate its specific biological role. Studies have reported that the disruption of PM structure facilitates the transport of the bacterial toxin into the midgut cells, enhancing microbial damage (136, 137). Disruption of chitin synthesis within the PM has been observed to impair the digestive processes in *S. litura*, resulting in reduced pupal weight and adult emergence rate (138). The complete inhibition of PM secretion by the chitin-binding reagent calcofluor has been found to induce larval developmental retardation, increased larval mortality rates, and a markedly increased susceptibility to baculoviral infection (139). *Bm01504*, one of many peritrophins discovered in silkworms, showed antiviral efficacy against BmNPV. The overexpression of *Bm01504* results in decreased expression of the key viral gene *p10*. Conversely, the RNAi of *Bm01504* resulted in increased expression of the *p10* viral gene, suggesting the potential antiviral role of PM against BmNPV (140). The protective effects of the PM matrix are also demonstrated in the other model insects like *T. castaneum*. In this context, the silencing of two PM matrix proteins, TcPMP3 and TcPMP5-B, via RNA interference has been correlated with a depleted fat body component and growth-related effects, ultimately leading to increased mortality rates in *T. castaneum* (131). Chitin Synthetase B (CHSB), encoded by the *CHS-2* gene, plays a pivotal role in chitin biosynthesis within the PM. RNAi targeting *CHS2* has been instrumental in elucidating its function, resulting in structural alterations within the mosquito’s PM. These alterations encompass vacuolization, cell invagination, partial cell rupture, and the conspicuous disruption of PM architecture (141). Pathogenic microbes, especially viruses, have evolved strategies to exploit the unique properties of the PM. They produce enhancins, which, in turn, manipulate the PM’s permeability (142, 143). Notably, the viral protein Chitinase A shares analogous functional attributes with enhancins, contributing to the degradation of PM integrity and the emergence of structural perforations when administered to silkworm larvae (144, 145). The Chitinase enzyme identified within *Spodoptera frugiperda* nucleopolyhedrovirus (SfMNPV) showed insecticidal properties through its interaction with the host’s PM (146, 147). A similar phenomenon occurs with *Dendrolimus kikuchii* nucleopolyhedrovirus, where the secretion of chitinase results in insecticidal activity via the degradation of the chitinous framework comprising the peritrophic membrane (148). Overall, the non-cellular PM is critical in preserving midgut structural integrity, facilitating the digestive processes, and acting as the first line of defense against pathogenic microbes. A deeper exploration of the functional roles of structural proteins within the PM promises to enhance our understanding of this intricate midgut component. Comprehensive studies remain imperative to unravel the multifaceted roles of peritrophins, particularly their discernible antiviral effects. These endeavors not only hold the potential to illuminate context-specific responses of the midgut to microbial challenges but also to unveil novel windows for fortifying immunity in economically significant organisms such as the silkworm. Furthermore, such investigations offer a promising route for identifying innovative targets in developing insecticides designed to control insect pest populations.

## 4 Midgut juice and immunity

The midgut juice, secreted by midgut cells (149) with a pH ranging from 9.2 to 11, has long been recognized for its digestive enzyme content essential for the digestion of food (24). **Table 3** includes the proteins from the midgut juice having immune characteristics. Studies have revealed the immunological role of digestive juice, unveiling the presence of proteins harboring potent antiviral functions (150). The antimicrobial properties of midgut juice were first reported by Hayashiya et al. They documented that the silkworm midgut juice has a distinctive red fluorescence emitting substance (150). Subsequent investigations have elucidated that a membrane protein, P252, which, upon binding with chlorophyllide, forms a red fluorescent protein (RFP) complex, displaying robust antiviral, antibacterial, and antifungal properties (151). Different forms of RFPs exhibit unique antiviral activities against different viruses (157). Interestingly, different silkworm varieties, including both resistant and susceptible strains (158), showed varying numbers of related RFPs, implying their involvement in the resistance mechanisms against pathogens. Silkworm midgut juice also contains other potent antiviral factors, including BmNOX (152), Bmlipase-1 (153) and serine protease-2 (155), all of which demonstrate strong antiviral activities. A 33% increase in the survival rate of transgenic silkworms challenged with BmNPV indicated the antiviral potential of Bmlipase-1 (154). The digestive enzyme trypsin, alkaline A (BmTA) also showed antiviral potential against BmNPV (47). Transcriptomic and proteomic analyses further highlight the significance of these proteins in the context of antiviral defense, with differential expression patterns observed in resistant and susceptible silkworms. However, despite the strong efficacy of these purified proteins as antiviral agents, the intricate mechanisms governing their antiviral activities still need to be discovered. Although modern multi-omics techniques have advanced our comprehension of the antimicrobial functions of silkworm midgut juice, comprehensive functional studies are required to unravel the precise molecular mechanisms underpinning these processes. This deeper understanding holds promise for enhancing our understanding of immunity against pathogens and opens new avenues for developing robust silkworm varieties, ultimately benefiting sericulture practices.

**Table 3:** Immunological characteristics of Midgut Juice.

Protein	Functions	Reference
Red Fluorescent protein	Antiviral activity upon binding with chlorophyllide	(150, 151),
BmNOX	Antiviral activity	(152)
Bmlipase-1	Antiviral activity	(153, 154),
Serine protease-2	Antiviral activity	(155)
Trypsin alkaline A (BmTA)	Antiviral activity	(47)
Lipase member H-A(BmLHA)	Antiviral activity	(156)

## 5 Conclusion and future perspectives

The complex relationship between silkworm midgut cell types and immunity has emerged as a fascinating area of research with far-reaching implications. The diverse range of midgut cells, including goblet, columnar, and enteroendocrine cells, play pivotal roles in the silkworm’s immune response. These cells collectively contribute to recognizing, signaling, and defense mechanisms against invading pathogens. The peritrophic membrane not only acts as a barrier but also serves as a platform for displaying immune-related molecules, aiding in detecting and neutralizing pathogens within the midgut lumen. The involvement of the midgut in pattern recognition, immune signaling, and the production of antimicrobial peptides and enzymes further shed light on the antimicrobial role of the second-largest organ of the insect.

Further investigation into the specific molecular mechanisms underlying the immune functions of different midgut cell types will provide a deeper understanding of how silkworms combat pathogens. High-throughput omics approaches, such as transcriptomics and proteomics, can unveil novel immune effectors. Knowledge based on these modern omics about the different cell types of the midgut will help to modulate the silkworm’s immune response through genetic manipulation or bioengineering, leading to enhanced disease resistance in silkworm populations, which has implications for silk production and sericulture. The antiviral potential of midgut juice and its components could be a platform for developing novel antiviral therapies for agricultural and medical purposes. Investigating how silkworm midgut immunity influences interactions with other organisms, including pathogens and gut symbionts, can shed light on broader ecological processes.

In conclusion, the intricate interplay between silkworm midgut cell types, the peritrophic membrane, and midgut juice in immunity open up a world of possibilities for both scientific exploration and practical applications, offering a promising path toward a deeper understanding of insect immune defenses and their potential for biotechnological advancements in sericulture and beyond.

## Author contributions

MA: Conceptualization, Writing – original draft. SF: Writing – original draft. JX: Writing – review & editing. MF: Conceptualization, Writing – review & editing. JS: Conceptualization, Funding acquisition, Supervision, Writing – review & editing.
